# Observation of interspecific feeding by White‐browed Shrike‐Babbler (*Pteruthius aeralatus*) of brood‐parasitic nestlings of Chestnut‐winged Cuckoo (*Clamator coromandus*) in a Nest of Greater Necklaced Laughingthrush (*Pterorhinus pectoralis*)

**DOI:** 10.1002/ece3.11465

**Published:** 2024-05-22

**Authors:** Wen Lu, Jinfa Li, Yuan Lei, Jingnan Duan, Kang Luo

**Affiliations:** ^1^ Administration Bureau of Nuozhadu Provincial Nature Reserve Puer Yunnan China; ^2^ State Key Laboratory of Environmental Geochemistry Institute of Geochemistry, Chinese Academy of Sciences Guiyang Guizhou China

**Keywords:** Chestnut‐winged Cuckoos, Greater Necklaced Laughingthrush, interspecific feeding, multiple parasitism, White‐browed Shrike‐Babbler

## Abstract

The occurrence of interspecific feeding events, involving non‐obligate nest parasite species, is rare but has been documented in numerous avian species worldwide, particularly in Europe and North America. Our report presents an observation from southwest China, where we observed a Greater Necklaced Laughingthrush (*Pterorhinus pectoralis*) nest containing three laughingthrush nestlings and two nestlings of Chestnut‐winged Cuckoos (*Clamator coromandus*). They were being fed by the adult laughingthrush and a male White‐browed Shrike‐Babbler (*Pteruthius aeralatus*). However, after the cuckoo nestling fledged, we did not observe the Shrike‐Babbler feeding the laughingthrush nestlings remaining in the nest. Through a systematic examination of potential driving factors, we infer that the begging calls of the cuckoo nestlings likely played a crucial role in the misfeeding events observed in our study. However, it is essential to consider the potential influence of the male shrike‐babbler's status, including mateless, brood loss or female incubation. We highlight the further observations using digital recordings (for both images and sounds) to document detailed information on interspecific feeding events.

## Graphical abstract

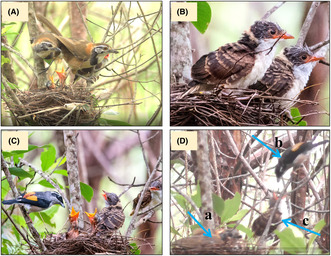

## 1 INTRODUCTION

Feeding of offspring in birds is usually conducted by one or both parents, but in some instances other individuals beside the biological parents help raising the young (Skutch, 1961). Intraspecific help is relatively common and cooperative breeding occurs in 9% of avian species (Cockburn, 1998; Griesser et al., 2017), providing benefits to bird populations, particularly in harsh environmental conditions (Cornwallis et al., 2017; Jetz & Rubenstein, 2011). However, interspecific feeding is an uncommon behavior where an individual of one species feeds individual (mostly offspring) of another species. This behavior has been observed among some fishes, mammals, and at least 2.4% of birds (Fiss et al., 2016; Skutch, 1961; Stacey & Koenig, 1990). For avian species, this behavior is more obvious and usually encountered in cases of obligate brood parasites, such as cuckoos (Cuculiformes) and cowbirds (Passeriformes: Icteridae) (Krüger & Pauli, 2017). Apart from cases involving brood parasite species, interspecific feeding in bird is rare, although this observation is relatively old and has been reported in ~41 bird families (Harmáčková, 2021; Shy, 1982).

Shy (1982) presented a taxonomy consisting of eight potential factors that may contribute to interspecific feeding. These factors include: (I) the presence of mixed clutches within a single nest, (II) the diversion of adult attention to other nests due to difficulties in finding a mate, (III) the loss of one's own clutch, (IV) the proximity of neighboring nests, (V) the presence of an incubating mate, (VI) the occurrence of loud calls from young individuals, (VII) the adoption of orphans, and (VIII) various other miscellaneous reasons. Based on Shy's findings, the last group (IX) was the most prevalent, accounting for 30% of the recorded instances. Close nests were found to be the second most common category, including 25% of the cases, while mixed clutches accounted for 22% of the cases (Harmáčková, 2021).

In contrast to the existing literature on intraspecific helping (Griesser et al., 2017; Koenig, 2017), there is a paucity of knowledge pertaining to the potential mechanisms of adaptation and evolutionary rationale underlying interspecific feeding. For example, a heightened drive to care for one's own young is generally advantageous, and feeding heterospecific young is possibly so rare that there is no significant evolutionary pressure to prevent this (Heber, 2013). Furthermore, although interspecific parental care provides little evolutionary benefit to the feeding bird, individuals engaging in interspecific provisioning behavior could benefit by increasing their parental abilities (Riedman, 1982; Shy, 1982; Trombino, 2000) if experience improves nesting success (de Steven, 1978; Lehrman & Wortis, 1967).

More occurrences of interspecific feeding should need to be documented, and it is crucial to provide detailed descriptions of the conditions underlying donor–recipient interactions (Harmáčková, 2021). Here, we are documenting the observations of Chestnut‐winged Cuckoo (*Clamator coromandus*) multiple parasitizing on Greater Necklaced Laughingthrush (*Pterorhinus pectoralis*), together with interspecific feeding assisted by White‐browed Shrike‐Babbler (*Pteruthius aeralatus*).

## 2 METHODS

Observations were made in a secondary Simao pine (*Pinus kesiya*) forest adjacent to the Nuozhadu Nature Reserve (NNR) (100°27′12″ E, 22°44′37″ N, 1438 m) in Yunnan Province, SW China. The NNR is positioned in the transitional zone between the north tropical and south subtropical zones, influenced by the southwest monsoon season, with an average annual temperature of 23°C and rainfall of 1400 mm. Notably, the NNR boasts rich biodiversity, encompassing 284 bird species, and has been designated as an Important Bird Area (IBA) by Birdlife International (BirdLife‐International, 2024).

**FIGURE 1:**
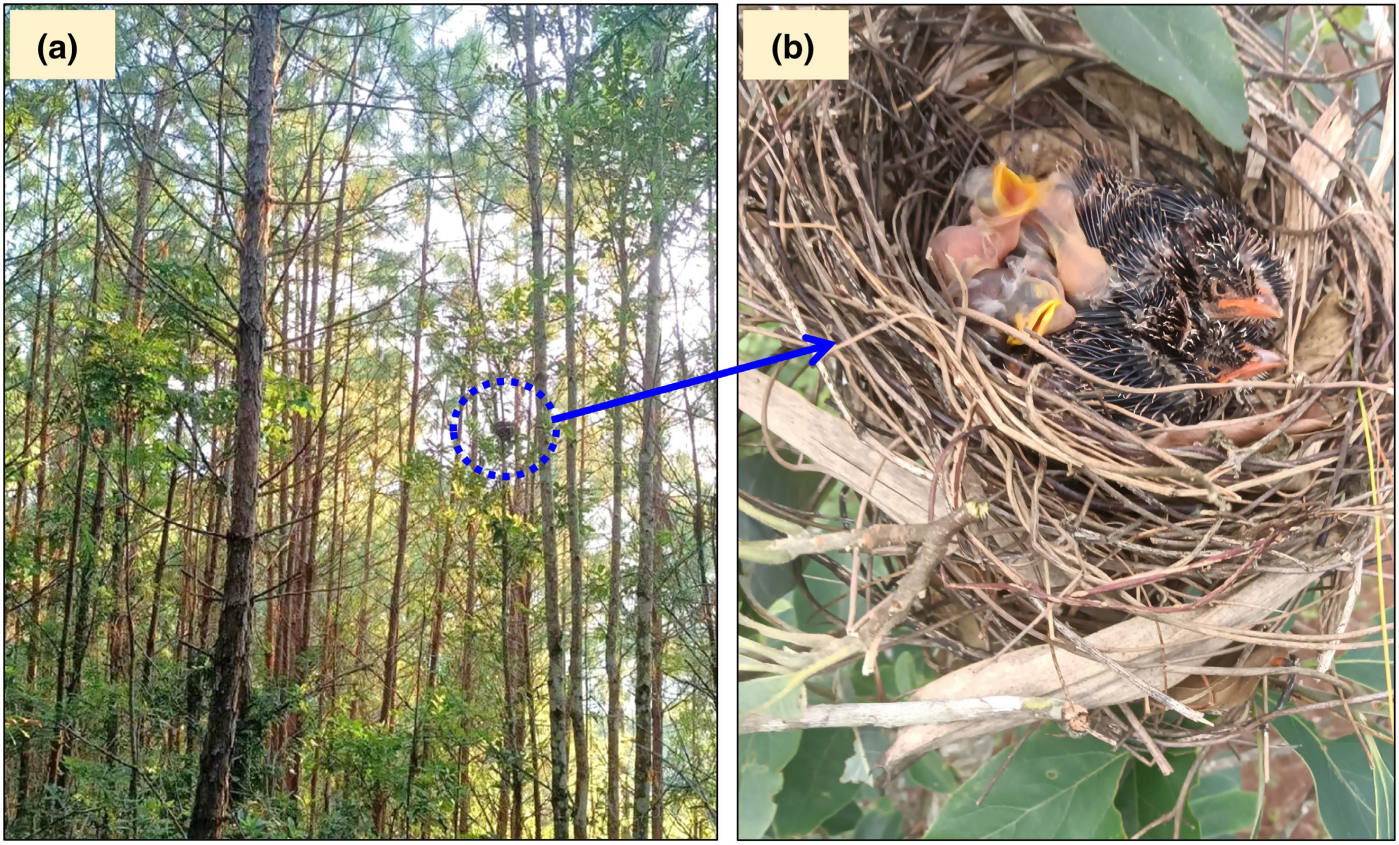
The microhabitat of the Greater Necklaced Laughingthrush nest site (a) and first appearance of the 5 nestlings in the nest (b) (Photo Credits: Cheng Wang).

A cup nest made with twigs and needles situated ~5 m above ground on the limbs of a tree (*Schima argentea*) was found on August 18, 2023 (Figure 1a). Upon discovery, the nest contained five nestlings. The five nestlings comprised two distinct types: two had a larger body size and more fully developed feathers, whereas the other three were hatchlings, identified by having a pink body dotted with a few downy feathers (Figure 1b). However, when the nest was re‐checked again on August 19, one of the smaller three nestlings (specifically, a laughingthrush nestling) had disappeared. Based on these feather and size development differences, the nest was assumed to be a case of brood parasitism.

Considering the nest site and cuckoo nestling development stage, there was a high risk of disturbing the cuckoo nestlings by climbing to install continuous recording devices (e.g., camera traps) near the nest, we chose to observe the nest instead. Therefore, we monitored the nest from August 18 until all the nestlings had fledged on September 1, using binoculars (i.e., Nikon, P3, 8 × 42) and digital cameras (i.e., Canon 50D; EF 400 mm f/4–5.6L IS USM) to record the parent‐care behaviors, from a cover ~15 m away from the nest. Given the remote location and practical limitations, we spent roughly 5 hours per day observing the parent‐care behaviors from August 20 to 24, totaling about 25 hours of observations (specific observation times were detailed in Figure 3). The nestlings did not fledge on the same day: the cuckoo nestlings departed the nest on August 21 and 22, respectively, while the last two laughingthrush nestlings fledged on September 1. Between August 25 and September 1, there was a lack of available volunteers, such as birdwatchers, which led us to rely on our forest rangers for monitoring the nest once or twice daily. Despite our diligent efforts, we failed to locate the helper's nest within approximately a 100 m radius of the laughingthrush nest and identify the fledged cuckoo nestlings in the nearby area due to the dense understory cover. Bird species was listed according to *Birds of the World* (https://birdsoftheworld.org/bow/home).

## 3 RESULTS

**FIGURE 2:**
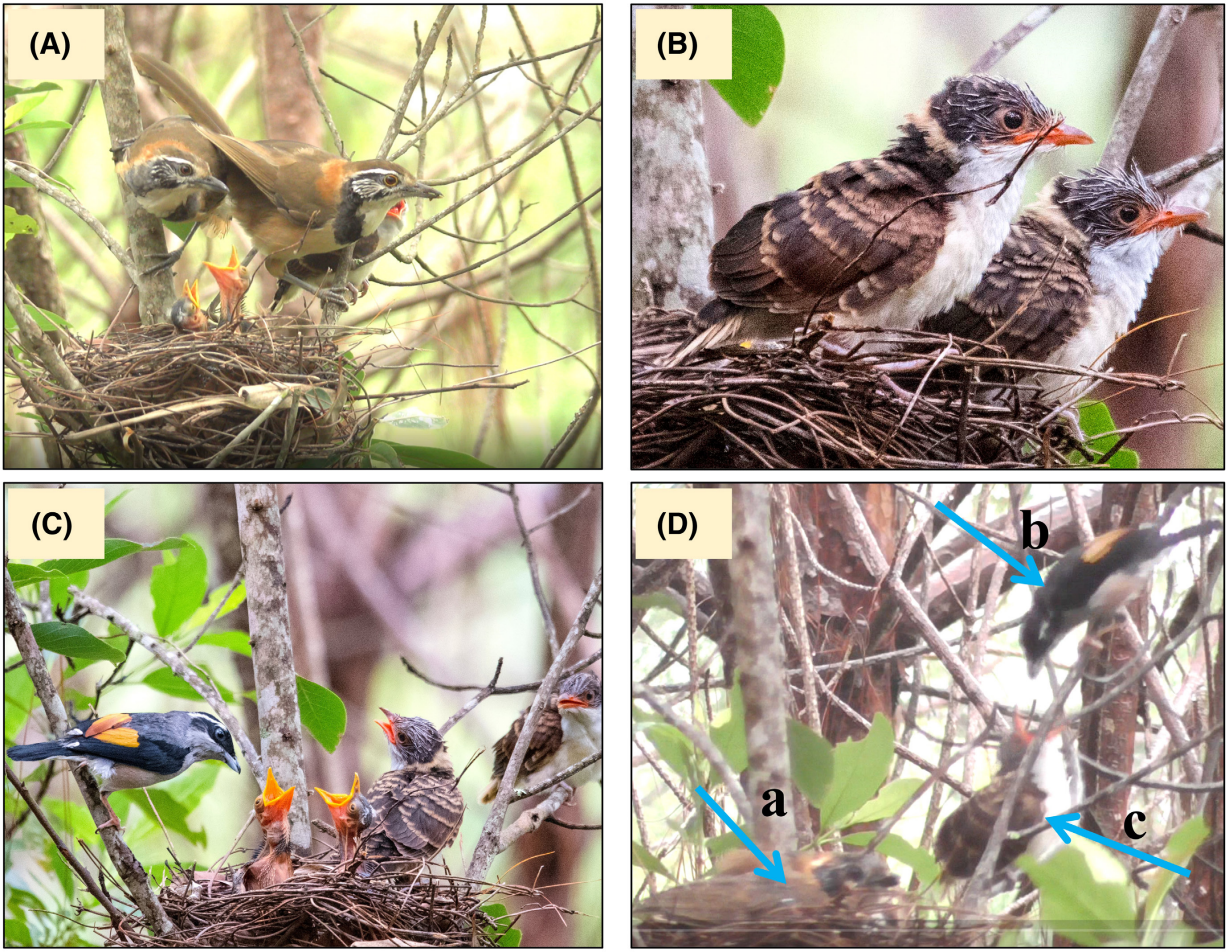
The laughingthrush adults feed the nestling (A), the appearance of Chestnut‐winged cuckoo nestling before fledging (B), the male White‐browed Shrike‐babbler showing up as a helper (C) and the encounter of laughingthrush adult (arrow‐a) and shrike‐babbler (arrow‐b) feeding the cuckoo nestling (arrow‐c) outside the nest (D) (Photo Credits: Rong Hu and Qiangxian Miao).

The nest belonged to a pair of Greater Necklaced Laughingthrush (*Pterorhinus pectoralis*; laughingthrush hereafter), which was confirmed by observations of both parents habitually showing up to feed and brood the nestlings (Figure 2A). Based on the visual appearance of the two bigger nestlings (Figure 2B) and the known distribution of cuckoo species at the research site, we confirmed that the nest was being parasitized by the Chestnut‐winged Cuckoo (*Clamator coromandus*; cuckoo hereafter). In addition, we observed a White‐browed Shrike‐Babbler (*Pteruthius aeralatus*; shrike‐babbler hereafter) that regularly showed up as a helper to feed both the laughingthrush and cuckoo nestlings (Figure 2C). Notably, the male Shrike‐babbler only helped at the nest during the period when the cuckoo nestling remained in the nest, that is, until August 22. Despite its occasional appearance around the nest territory (~5 m away from the nest), we did not record any instances of the male shrike‐babbler feeding the laughingthrush nestlings during observation on August 23 and 24. In addition, we failed to locate the cuckoo fledglings due to the *in suit* dense understory cover.

**FIGURE 3:**
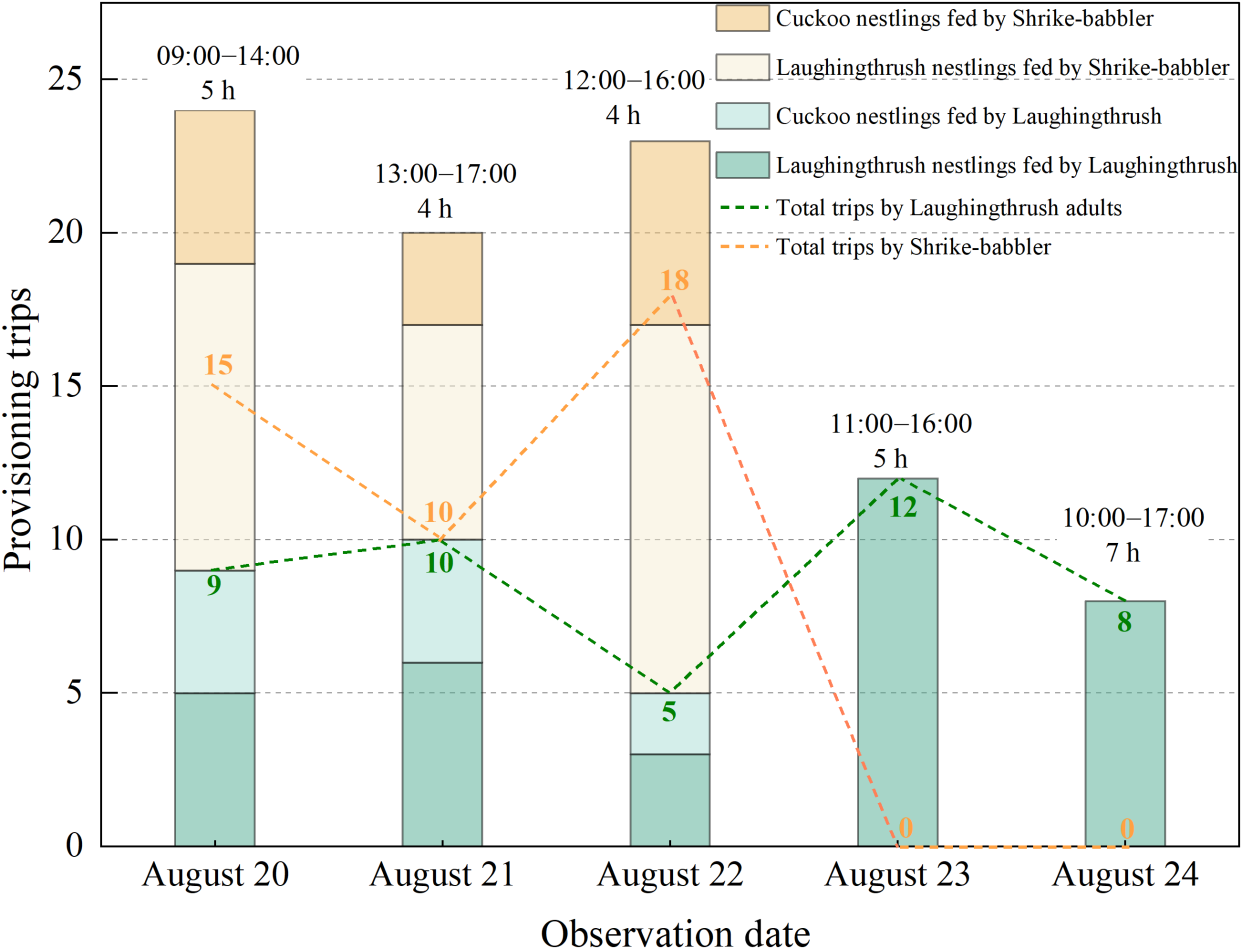
The feed trips by laughingthrush adults and shrike‐babbler to laughingthrush and cuckoo nestlings during the observation period. The digits above the bars represent the observation period (UTC + 8) and the total observation hours per day, respectively.

We counted 87 provisioning events in all (Figure 3), 44 of which were by the adult laughingthrushes and 43 of which were by the male shrike‐babbler. The laughingthrush adults fed the cuckoo nestlings 10 times, while the male shrike‐babbler fed them 14 times. Moreover, the male shrike‐babbler fed the laughingthrush nestlings 29 times, and the laughingthrush adults fed their own nestlings 34 times during the observation period. The male shrike‐babbler adult and the laughingthrush adults both removed fecal sacs from the nest after feeding if the nestlings defected, but the precise number of instances of fecal removal could not be counted.

Notably, on August 22 the adult laughingthrush brooded the nest due to the rainy weather, and the second cuckoo nestling perched on the limb adjacent to the nest. The male shrike‐babbler adult arrived and fed the cuckoo nestling outside the nest appropriately. The laughingthrush adult noticed this but did not behave violently to scare the male shrike‐babbler away from the cuckoo nestlings (Figure 2D). The male shrike‐babbler did not attempt to feed the cuckoo nestling remaining in the nest during this period.

## 4 DISCUSSION

To the best of our knowledge, this is the first report of interspecific feeding to the Greater Necklaced Laughingthrush nestlings or Chestnut‐winged Cuckoo nestling by the White‐browed Shrike‐Babbler (Harmáčková, 2021; Shy, 1982). Our observation also provides a case study of parasitism by the Chestnut‐winged Cuckoo on the Greater Necklaced Laughing Thrush involving multiple cuckoo nestlings. The combination of these two observations occurring concurrently in one nest is even more noteworthy (Harmáčková, 2021, Shy, 1982).

The fact that only the male Shrike‐babbler showed up as a helper supports the hypothesis that males are more frequent helpers than females, possibly due to having more time and opportunities (Cockburn, 1998; Shy, 1982). Several factors have been considered to explain the cause of the misfeeding events by the helper (Shy, 1982). In our case, the possibility of Factor IV (the proximity of neighboring nests) in driving the interspecific feeding events can be ruled out, as there was no shrike‐babbler nest in close proximity to the laughingthrush nest. The laughingthrush nest contained a mix of laughingthrush and cuckoo nestlings, but no shrike‐babbler nestlings, therefore Factor I (mixed clutches within a single nest) is also not the primary factor (Harmáčková, 2021). The nestlings (both laughingthrush and cuckoo) being raised by the laughingthrush parents makes Factor VII (the adoption of orphans) unlikely in our case. Since we did not ascertain the helper's status, we cannot definitively determine whether Factor II (difficulties in finding a mate), Factor III (the loss of its own clutch) and Factor V (the presence of the incubating mate) were the main driving factors.

In addition, our observations suggest that the loud begging calls of the cuckoo nestlings (Factor VI: the occurrence of loud calls from young individuals) may have played a significant role in this misfeeding events to some extent (Schaeffer et al., 2009; Sealy & Lorenzana, 1997). This is supported by the observation that when the cuckoo nestlings were present in the nest between August 20 and 22, there were overall louder begging calls. Concurrently, the cuckoo nestlings also displayed more vigorous movements in acquiring (and at times, pilfering) food items from the provisioning parents, in contrast to when only the laughingthrush nestlings were present on August 23 and 24. Additionally, the shrike‐babbler made more provisioning trips to the nest during August 20–22 than the laughingthrush adults did during the same period (43 trips versus 24 trips). However, although the male shrike‐babbler showed up near the nest vicinity a few times during the observations between August 23 and 24, it did not feed the remaining two laughingthrush nestlings in the nest. Drawing from the observation that the male shrike fed the cuckoo nestling while it was perched on a branch ~10 cm away from the nest before fledging (Figure 2d), we speculate that the male shrike‐babbler may have been lured to continue to feed the cuckoos fledglings nearby (Sealy & Lorenzana, 1997; Tyller et al., 2018). It is essential to acknowledge that our inference regarding the role of begging calls in driving interspecific feeding is constrained by the absence of direct acoustic analysis of the begging calls (Batisteli & Sarmento, 2016). For future studies encountering the interspecific feeding events, we highly recommend recording the begging calls for detailed acoustic analysis.

The development of monitoring devices has facilitated the acquisition of detailed information from observations on interspecific feeding cases (Harmáčková, 2021; Jiang et al., 2016). For instance, the removal of fecal pellets by the helper, which could have strong implications for nest safety from predation (Guigueno & Sealy, 2011), was not mentioned in older literatures (Shy, 1982; Skutch, 1961), but has been increasingly reported in recent publications (Harmáčková, 2021; Jiang et al., 2016; Luo et al., 2018). Continuous digital videos and photos have also provided detailed insights into parental care at the nest, including food item compositions and provisioning rates (Batisteli & Sarmento, 2016; Luo et al., 2018).

In the encounters between the laughingthrush adult and the shrike‐babbler in the nest vicinity described above, we observed no signs of aggression or violent behavior in either of the adult birds. This suggests that the laughingthrush adult may have calmly accepted the help of the shrike‐babbler, as observed in other interspecific feeding cases in Southwest China (Luo et al., 2018). This behavior contrasts with situations where nest owners chase away the helper (Schaeffer et al., 2009), or where the helper takes charge of the nestlings after a confrontation (Haucke, 2015). The submissive feeding behavior observed may suggest that the helper is aware that they are approaching the wrong nest or vicinity, indicating a recognition of their mistaken behavior. This raises the possibility that these helping actions are not simply errors or misfeeding events, but rather a behavior that the helper cannot refrain from. However, this intriguing behavior warrants further investigation, and detailed observations with digital recordings (for both images and sounds) are essential to unravel the complexities of interspecific feeding events and the underlying motivations driving such behavior.

## AUTHOR CONTRIBUTIONS

**Wen Lu:** Conceptualization (equal); formal analysis (equal); funding acquisition (equal); methodology (equal); project administration (supporting); supervision (supporting); validation (equal); writing – original draft (equal); writing – review and editing (supporting). **Jinfa Li:** Conceptualization (equal); formal analysis (equal); funding acquisition (equal); investigation (lead); methodology (equal); project administration (lead); validation (equal); visualization (supporting); writing – original draft (supporting); writing – review and editing (supporting). **Yuan Lei:** Investigation (supporting); writing – original draft (supporting). **Jingnan Duan:** Methodology (supporting); validation (equal). **Kang Luo:** Formal analysis (equal); visualization (lead); writing – original draft (equal); writing – review and editing (lead).

## FUNDING INFORMATION

This study was funded by Nuozhadu Nature Reserve for Endangered Species Monitoring, Observation and Research Station of Yunnan Province (Grant/Award Number: 202205AM070003) and the National Natural Science Foundation of China (Grant/Award Number: 42207311).

## CONFLICT OF INTEREST STATEMENT

The authors declare no conflicts of interest.

### OPEN RESEARCH BADGES

This article has earned Open Data and Open Materials badges. Data and materials are available at https://datadryad.org/stash/share/NiuBGl_b2mO_ouU3DW5m7j3Q_inxaBRqHm_BONERCr8.

## Data Availability

The data (video recordings and observation details) supporting this study's findings are openly available in Dryad at https://doi.org/10.5061/dryad.547d7wmgs.
